# Absolute and relative physical activity intensity thresholds across diverse fitness levels: a cross-sectional study in middle-aged women

**DOI:** 10.3389/fspor.2026.1829167

**Published:** 2026-07-06

**Authors:** Jonatan Fridolfsson, Andreas Malmgren, Petri Gudmundsson, Magnus Dencker

**Affiliations:** 1 Center for Lifestyle Intervention, Institute of Medicine, Sahlgrenska Academy, University of Gothenburg, Gothenburg, Sweden; 2 Department of Medicine, Geriatrics and Emergency Medicine Östra, Sahlgrenska University Hospital, Gothenburg, Sweden; 3 Department of Translational Medicine, Lund University, Malmö, Sweden; 4 Department of Medical Imaging and Physiology, Skåne University Hospital, Malmö, Sweden; 5 Department of Biomedical Science, Faculty of Health and Society, Malmö University, Malmö, Sweden; 6 Biofilms Research Centre for Biointerfaces, Malmö University, Malmö, Sweden

**Keywords:** accelerometry, aerobic fitness, exercise, female, physical activity, sports

## Abstract

**Introduction:**

Physical activity (PA) intensity can be expressed in absolute terms (metabolic equivalents, METs) or relative to individual capacity (percentage of maximal oxygen consumption, VO₂max), and these two approaches yield markedly different interpretations of health-related PA. This study examined how time spent at absolute and relative PA intensities relates to cardiorespiratory fitness (CRF) in middle-aged women across a wide fitness range.

**Methods:**

In this cross-sectional study, 73 middle-aged women (38 recreational runners, 35 inactive controls) aged 50-65 years with diverse CRF levels (20-60 mL/min/kg) were assessed. CRF was assessed through maximal cycle ergometer testing. PA was measured via hip-worn accelerometry for seven days using the 4 Hz frequency extended method. Absolute intensity used traditional METs thresholds (3-6 METs moderate, ≥6 METs vigorous); relative intensity was expressed as percentage of individual VO₂max (46-64% moderate, ≥64% vigorous). Associations with CRF were analysed using partial least squares regression and cumulative intensity thresholds.

**Results:**

The active group had significantly higher CRF (41.4 vs. 27.7 mL/min/kg, *p* < 0.001) and accumulated substantially more vigorous activity (20.6 vs. 0.8 min/day, *p* < 0.001), whereas the inactive group accumulated more relative moderate intensity, illustrating the “intensity paradox.” For absolute intensity, positive CRF associations emerged at approximately 4.5 METs, strengthening progressively and peaking in the vigorous range (>6 METs). For relative intensity, negative associations diminished progressively, reaching equilibrium near 64% VO₂max. The relative intensity model explained 70% of CRF variance compared to 53% for absolute intensity.

**Discussion:**

In this cross-sectional sample, traditional 3 METs moderate intensity thresholds appear inadequate for middle-aged women, with significant CRF associations occurring primarily at vigorous intensity. These findings suggest that vigorous intensity should be the primary target when CRF is the desired outcome, and support prescribing intensity relative to individual capacity.

## 1 Introduction

Physical activity (PA) intensity is critical for understanding the relationship between PA and health (1). However, measuring and interpreting PA intensity remains complex when using accelerometer-based assessments that translate mechanical acceleration into physiologically meaningful metrics (2). The distinction between absolute and relative PA intensity is fundamental in research and clinical practice, yet these approaches yield markedly different interpretations of health-related PA (3, 4). While physical activity at all intensities confers health benefits including improved glucose metabolism, bone density, and mental health (1), the present study focuses specifically on cardiorespiratory fitness (CRF) as the health outcome, given its strong dose-response relationship with mortality and its direct link to exercise intensity (5).

Absolute PA intensity measures energy expenditure in standardized units, typically metabolic equivalents of task (METs), where moderate intensity is defined as 3–6 METs regardless of individual CRF (1, 6). Relative PA intensity expresses activity as a percentage of individual maximal oxygen consumption (VO_2_max), with moderate intensity defined as 46%–64% of VO_2_max (6). While absolute intensity represents the same energetic demand across individuals, relative intensity reflects physiological strain based on CRF level (1, 6).

Accelerometers measure PA through body acceleration, calibrated against absolute intensity thresholds like 3 METs (2, 7). This calibration assumes a uniform relationship between acceleration and metabolic demand across individuals, overlooking substantial CRF variation. Current PA guidelines assume 3 METs represents moderate relative intensity for most adults. However, 3 METs only corresponds to 46% of VO_2_max (the lower bound of relative moderate intensity) for individuals with CRF of 22.8 mL/min/kg (since 3 METs = 3·3.5 mL/min/kg = 10.5 mL/min/kg, and 10.5/0.46 ≈ 22.8). This level is substantially lower than population averages (3, 8, 9). For typical middle-aged adults [VO_2_max 33 mL/min/kg (10)], 3 METs represents only 30% of maximal capacity, falling within light intensity. This means the widely used 3 METs threshold is too low for 95% of middle-aged adults, potentially causing systematic misclassification (3).

Individualized calibration studies show that relative moderate intensity typically corresponds to 4–5 METs, substantially higher than traditional thresholds (8, 9). This creates an “intensity paradox” wherein low-fit individuals appear most active using relative measures but least active with absolute measures (11). Recent evidence suggests relative intensity better predicts intervention responses (12) and shows distinct mortality associations varying by sex (4). Population-based approaches using age- and sex-specific thresholds have emerged as practical alternatives to individual calibration (13, 14), while methods combining both absolute and relative metrics provide enhanced insights (15).

Previous research examining absolute vs. relative PA intensity has faced three critical limitations. First, most studies included participants with narrow CRF ranges, limiting examination of PA-health associations across the CRF spectrum (3, 4). Second, most relied on submaximal CRF tests rather than maximal exercise testing, potentially introducing systematic error (16), particularly at higher CRF levels. Third, previous studies typically examined general population samples divided into CRF quantiles rather than comparing individual activity patterns.

This study examines how absolute and relative physical activity intensity relate to CRF in middle-aged women with diverse CRF levels using partial least squares regression, cumulative intensity thresholds, and maximal cycle ergometer testing. We hypothesized that: 1. the active group would accumulate more absolute physical activity overall than the inactive group; 2. when intensity was expressed in relative terms, the lower bound of the positive PA–CRF association would shift upward from the traditional 3 METs moderate threshold to a higher intensity; and 3. light and lower-moderate relative intensities would show negative associations with CRF, consistent with the intensity paradox previously described in low-fit individuals. From an applied perspective, the disconnect outlined above between the absolute (3 METs) and relative (46% VO_2_max) moderate-intensity thresholds means that the two metrics rarely identify the same activity as moderate within a given individual. Identifying which of the two carries the CRF signal would therefore clarify which threshold should anchor exercise prescription in sports science and clinical exercise physiology.

## 2 Materials and methods

This cross-sectional study investigated absolute and relative physical activity intensity patterns and their associations with CRF in Swedish middle-aged women with diverse CRF levels.

### 2.1 Study sample

This study was approved by the Regional Ethical Review Board in Lund (no. 2015/504) and was performed in accordance with the ethical standards in the 1964 Declaration of Helsinki. All participants provided written informed consent. Recruitment and measurements occurred April 2016-November 2017. Patients or members of the public were not involved in the design, conduct, reporting, or dissemination plans of this research.

Middle-aged women were chosen as the target population for three reasons. First, this group is underrepresented in accelerometer-based studies of CRF, which have mostly been conducted in mixed-sex or male-dominated cohorts. Second, established collaborations with sports clubs and an available population register sample in the Malmö-Lund region provided the practical basis for parallel recruitment of recreational athletes and inactive women in the same age band. Third, contrasting these two extremes within a single age- and sex-homogeneous group was the most efficient way to obtain the wide CRF range required to characterize PA-CRF associations across the physiological spectrum.

The study sample comprised middle-aged women aged 50–65 years. Exclusion criteria included known cardiovascular disease, diabetes, cancer, previous treatment for serious diseases (including radiation therapy or chemotherapy), and current smoking or smokeless tobacco use. Participants were recruited through two strategies targeting diverse physical activity patterns.

The active group consisted of recreational endurance athletes recruited through local sports clubs predominately in the Malmö-Lund region, Sweden. Clubs specializing in endurance sports (running, orienteering, cycling, triathlon) were contacted via email or telephone. Group-specific inclusion required regular training and competition for at least 2 years and at least 4 h of organized training per week.

The inactive group was recruited through random selection from the population register based on sex, age, and residence. Invitation letters were mailed to 2,000 potential participants across four occasions. Group-specific inclusion required no organized training or systematic exercise for at least 5 years.

**Figure 1:**
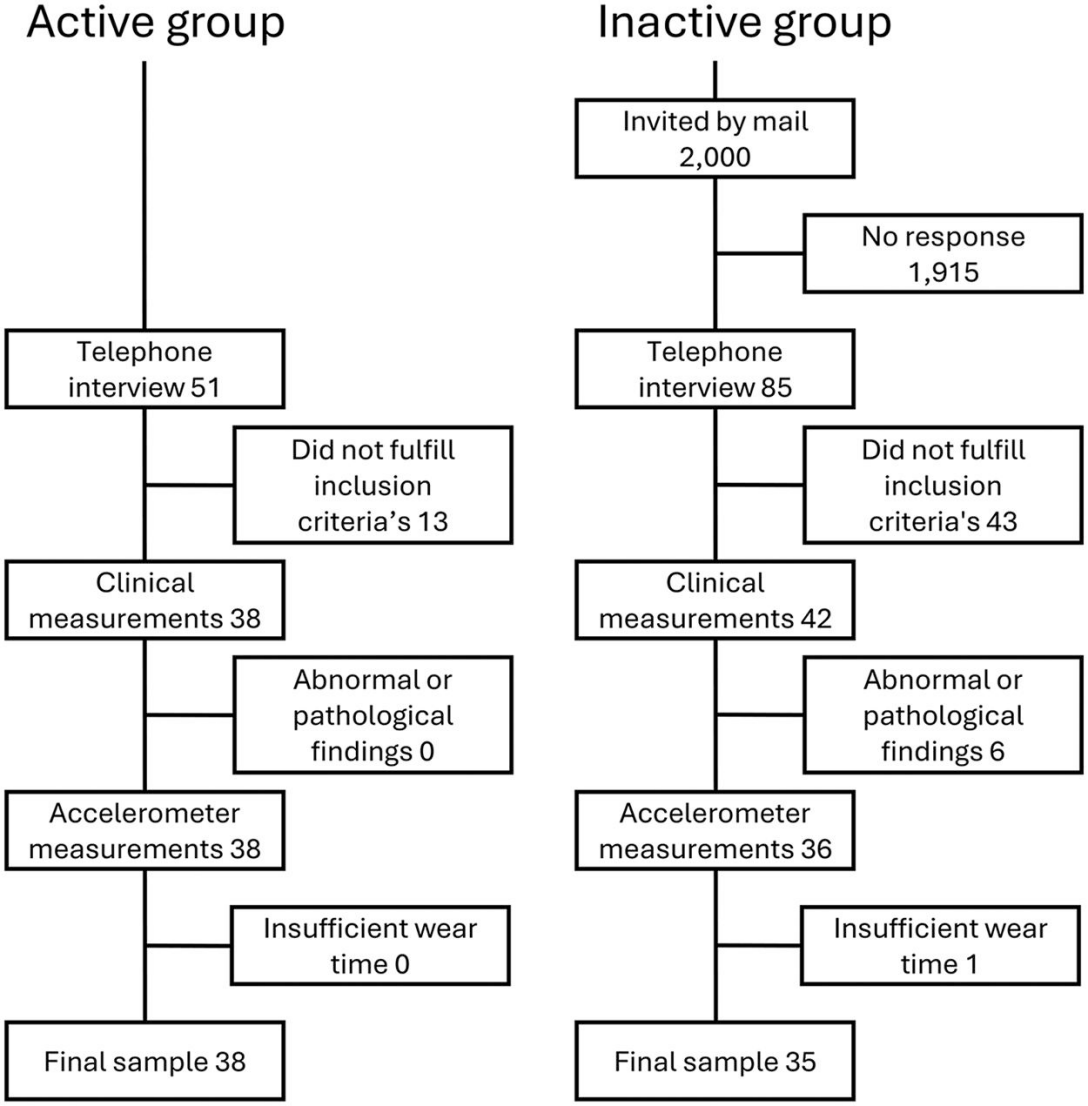
Flowchart of sample recruitment.

Following telephone screening and clinical measurements, six participants from the inactive group were excluded due to abnormal laboratory results or pathological findings, and one for insufficient accelerometer wear time. No participants from the active group were excluded. The final sample consisted of 73 middle-aged women: 38 active and 35 inactive (Figure 1). Sample size was determined to provide adequate statistical power for partial least squares regression analyses, targeting a minimum of 25–30 participants per group within the available recruitment period. This sample size (*N* = 73) provides > 80% power to detect bivariate correlations of r ≥ 0.32 at *α* = 0.05 (calculated using the standard correlation power formula), adequate for the large effect sizes anticipated in CRF associations.

### 2.2 Cardiorespiratory fitness

CRF was estimated using a progressive maximal cycle ergometer test on an electronically braked ergometer (Ergomedic 939E, Monark Exercise AB, Vansbro, Sweden). Participants started at 50 W with workload increasing by 15 W per minute until volitional exhaustion while maintaining 60–65 rpm cadence (17). Maximal power output (Wpeak) included the last completed workload plus a fraction of any partial stage. Maximal oxygen consumption was estimated, rather than directly measured through gas exchange analysis, using a validated equation: VO_2_max (L·min^−1^) = 0.0117 × Wpeak (W) + 0.16 (18), and expressed relative to body weight (mL/min/kg). This equation explains 80% of VO_2_max variability (r = 0.88) with test-retest correlations of 0.95–0.96 (18).

### 2.3 Physical activity

Physical activity was measured using triaxial accelerometers (ActiGraph GT3X+, Pensacola, Florida, USA) worn over the right hip for seven consecutive days, removed only during water-based activities. Raw data were processed using the 4 Hz frequency extended method with 1-second epochs (19, 20), which provides superior capture of health-related physical activity compared to traditional count-based approaches (19). Non-wear time was defined as 60 consecutive minutes of zero output, allowing up to 2 min of interruptions below sedentary threshold (21). Valid measurements required at least 4 days with ≥ 10 h wear time (7).

Processed accelerometer output (vector magnitude in mg from the 4 Hz frequency extended method) was divided into a comprehensive intensity spectrum consisting of 22 intensity variables with bin edges at 0, 25, 50, 100 mg, then 50 mg intervals up to 1,000 mg and above. All intensity spectrum variables are expressed as minutes per day. Traditional intensity categories were calculated based on previous calibration (20): sedentary (< 30.8 mg), light physical activity (LPA, 30.8-< 133.7 mg), moderate physical activity (MPA, 133.7-< 498.1 mg), vigorous physical activity (VPA, 498.1-< 881.2 mg), and very vigorous physical activity (VVPA, ≥ 881.2 mg). These spectrum- and traditional category variables represented absolute intensity.

For relative intensity analysis, the individually measured VO_2_max from the cycle ergometer test was converted to a person-specific accelerometer ceiling using a linear calibration line fitted between the absolute cut-points and their corresponding metabolic equivalents (1.5, 3, 6 and 9 METs at 30.8, 133.7, 498.1 and 881.2 mg, respectively). The bin definitions were applied in MATLAB to the same processed mg time-series. The mg range from zero to the participant's VO_2_max-derived ceiling was then divided into an additional 27-bin spectrum from 7% to 100% (3, 20). Relative intensity cut-points were defined as: light (14% - < 46% VO_2_max), moderate (46% - < 64% VO_2_max), vigorous (64% - < 91% VO_2_max), and very vigorous (≥ 91% VO_2_max) 6.

### 2.4 Statistical analysis

All participants with valid physical activity measurement were included in the study and no imputation was required. Group differences in characteristics and traditional PA variables were assessed using independent samples t-tests. Normality of CRF was assessed using the Lilliefors test. Significance was set at *p* < 0.05.

Partial least squares (PLS) regression was employed for intensity spectrum analysis due to inherent multicollinearity in 24-hour movement behavior (22). PLS effectively handles collinear variables by decomposing explanatory variables into linear combinations while maximizing covariance with the outcome (23). For CRF associations, PLS regression examined relationships between the detailed PA intensity spectrum and CRF. For group discrimination, PLS discriminant analysis (PLS-DA) identified PA patterns distinguishing active and inactive groups (24). All variables were z-score standardized prior to analysis. Model validation used Monte Carlo resampling with 1,000 repetitions, randomly selecting 50% as external validation. Component number was determined using cross-validation with a cut-off of one-quarter standard deviation (25).

Physical activity patterns were visualized using multivariate correlation plots representing standardized associations between each intensity variable and the outcome while accounting for multicollinear structure 22. Multivariate correlations were derived from selectivity ratios by taking the square root and applying appropriate sign. Confidence intervals (95%) were obtained through bootstrapping (10,000 repetitions).

Cumulative cut-point analysis calculated correlations and unstandardized regression coefficients between CRF and cumulative time above progressively higher intensity thresholds. This dual approach identifies optimal intensity thresholds while providing both statistical significance and clinical relevance. Bootstrap confidence intervals (10,000 repetitions) were calculated for all coefficients.

All analyses were performed using MATLAB 2024b (MathWorks, Natick, MA, USA).

The manuscript text was edited for clarity and language using the AI based large language model Claude 4.5 Sonnet (Anthropic, San Francisco, CA, USA). The AI model was not involved in study design, data collection, data analysis, interpretation of results, or generation of scientific content. The authors reviewed and verified all AI-assisted edits and take full responsibility for the final content.

## 3 Results

### 3.1 Sample characteristics

**Figure 2:**
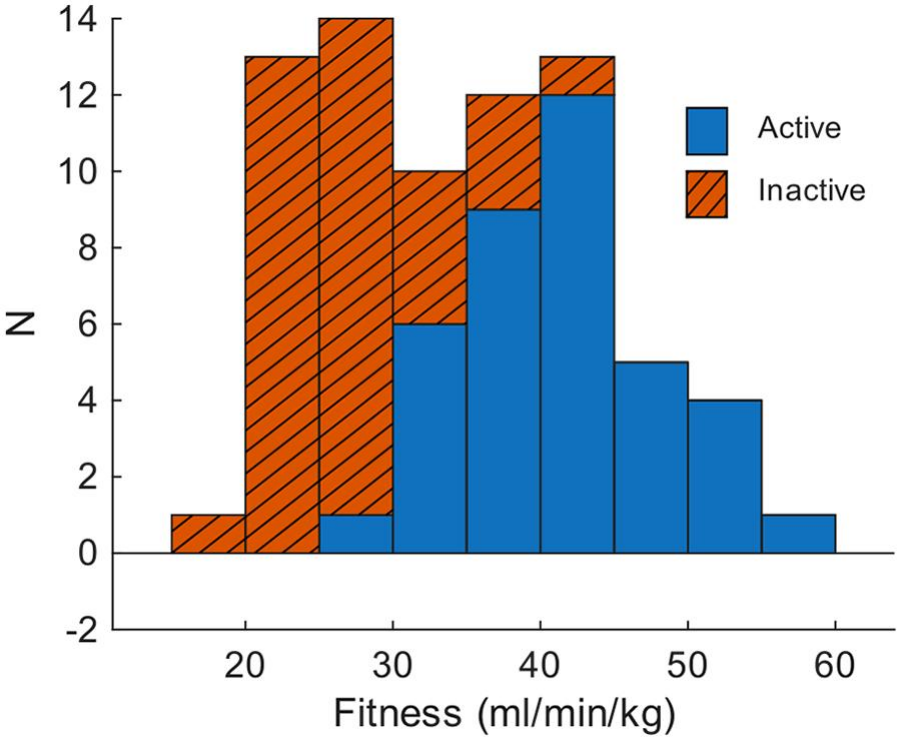
Distribution of fitness by activity group and in the overall sample.

The study included 73 middle-aged women (mean age 57.5 years). The active (*n* = 38) and inactive (*n* = 35) groups showed no age difference but differed significantly in CRF (41.4 vs. 27.7 mL/min/kg, *p* < 0.001), BMI (22.1 vs. 25.0 kg/m^2^, *p* < 0.001), and self-reported weekly training volume (7.1 vs. 0.1 h, *p* < 0.001). Table 1 presents descriptive statistics. The combined CRF distribution was approximately normal (Lilliefors test, *p* = 0.13), ranging from 20 to 60 mL/min/kg (Figure 2).

**Table 1:** Descriptive statistics of the overall sample and the active and inactive group.

Characteristic	Overall	Active group	Inactive group
Age (years)	57.5 (3.5)	57.3 (3.5)	57.8 (3.5)
BMI	23.5 (3.5)	22.1 (2.3)	25.0 (3.9)*
Self reported training (h/week)	3.75 (4.03)	7.14 (2.62)	0.06 (0.26)
Fitness (mL/kg/min)	34.81 (9.3)	41.37 (7.0)	27.69 (5.5)*
Absolute physical activity	(min/day)		
Sedentary	1,195.5 (51.7)	1,180.0 (49.7)	1,212.3 (49.2)*
Light	131.1 (28.2)	134.9 (26.2)	127.1 (30.1)
Moderate	102.3 (26.7)	104.5 (27.7)	99.9 (25.7)
Vigorous	11.1 (15.2)	20.6 (16.1)	0.8 (0.8)*
Relative physical activity	(min/day)		
Light	210.05 (55.4)	235.52 (47.2)	182.40 (50.6)*
Moderate	25.06 (21.8)	14.21 (9.7)	36.84 (25.2)*
Vigorous	9.41 (11.0)	10.25 (10.2)	8.51 (11.9)

Mean (standard deviation).

* Denotes significant group difference at *p* < 0.05.

### 3.2 Group differences in PA intensity

**Figure 3:**
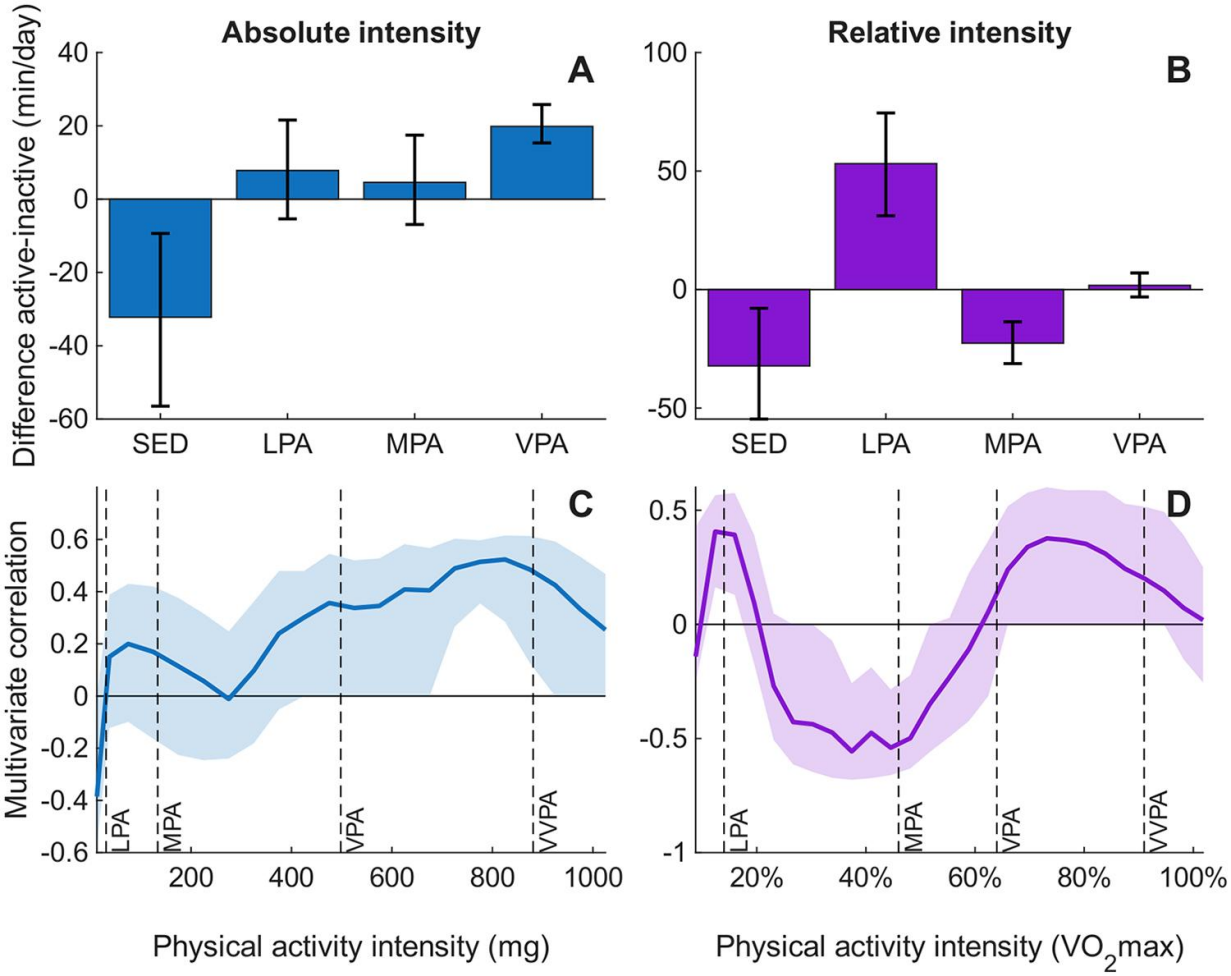
Group comparison of absolute and relative intensity physical activity. **(A,B)** Traditional cut-point analysis showing differences between active and inactive groups for sedentary (SED), light physical activity (LPA), moderate physical activity (MPA), and vigorous physical activity (VPA). **(C,D)** Multivariate pattern analysis across the intensity spectrum, with dashed lines indicating thresholds for LPA, MPA, VPA, and very vigorous physical activity (VVPA).

PLS discriminant analysis (one component retained for all models) distinguished between groups using both absolute (R^2^ = 0.47, *p* < 0.01, classification accuracy 84.9%) and relative intensity (R^2^ = 0.58, *p* < 0.01, 86.3%), with relative intensity providing slightly superior discrimination. For absolute intensity, the active group accumulated significantly more vigorous activity (20.6 vs. 0.8 min/day, *p* < 0.001) and less sedentary time (1,180.0 vs. 1,212.3 min/day, *p* = 0.007), with no differences in light or moderate intensity (Figure 3A). For relative intensity, the active group accumulated more relative light intensity (235.5 vs. 182.4 min/day, *p* < 0.001) while the inactive group accumulated more relative moderate intensity (36.8 vs. 14.2 min/day, *p* < 0.001), with no difference in vigorous intensity (Figure 3B).

Multivariate patterns showed that for absolute intensity, the active group performed increasingly more activity from mid-moderate intensity, peaking in upper vigorous range (Figure 3C). For relative intensity, the active group performed more activity in lower light intensity but less in upper light and moderate intensity ranges, with a tendency toward more relative vigorous intensity (Figure 3D).

### 3.3 Cardiorespiratory fitness associations across the intensity spectrum

**Figure 4:**
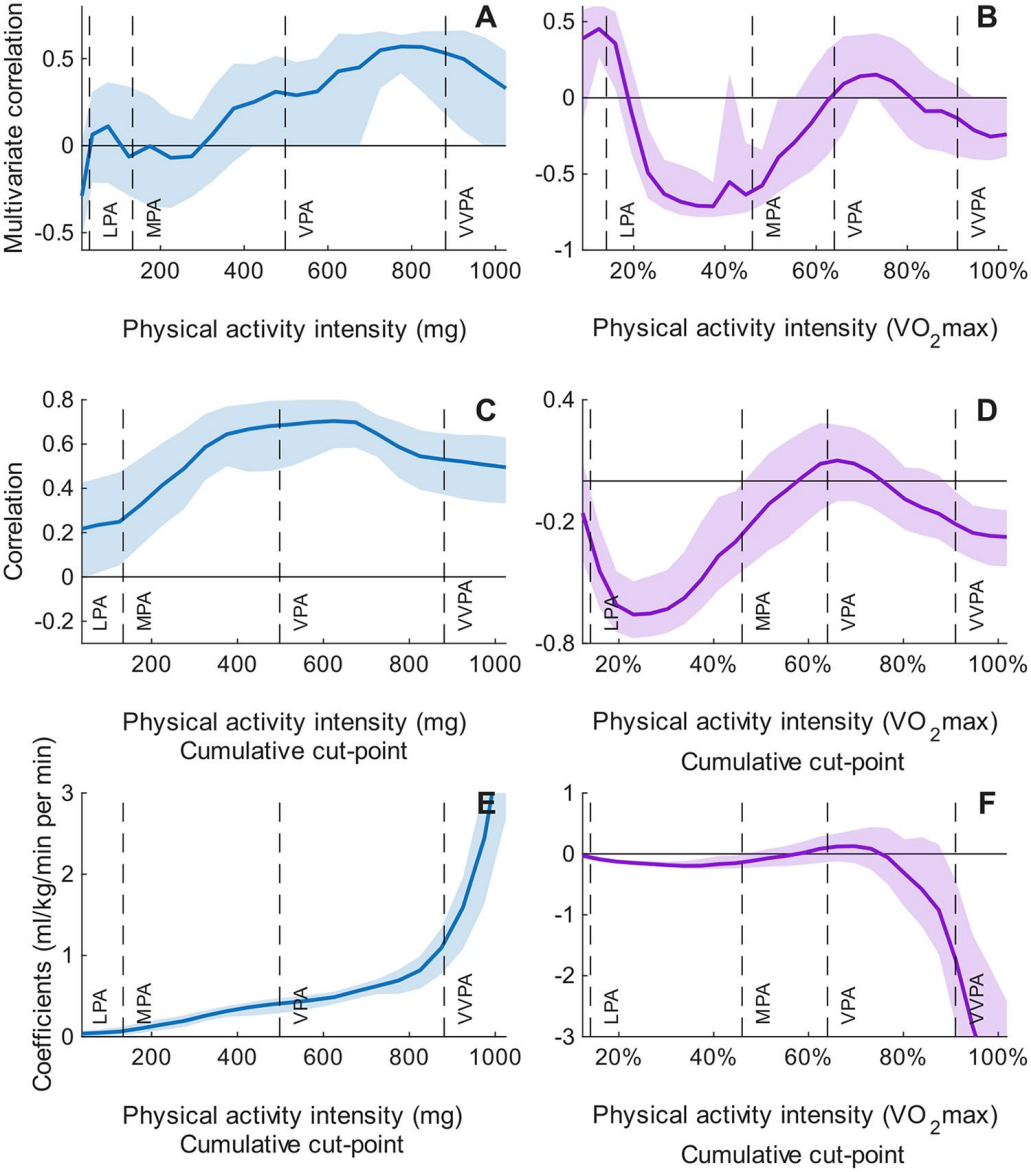
Absolute and relative intensity physical activity patterns related to fitness in the overall sample. Dashed lines indicate thresholds for light physical activity (LPA), moderate physical activity (MPA), vigorous physical activity (VPA), and very vigorous physical activity (VVPA). **(A,B)** Multivariate correlations across intensity spectrum. **(C,D)** Cumulative correlations showing associations with time spent above progressively higher intensity thresholds. E-F: Cumulative regression coefficients (mL/kg/min per min physical activity) quantifying fitness associations.

PLS regression examining CRF associations yielded significant models for both absolute (R^2^ = 0.53, *p* < 0.01) and relative intensity (R^2^ = 0.70, *p* < 0.01). For absolute intensity, positive associations emerged in mid-moderate range (∼ 4.5 METs), strengthening progressively and peaking in the vigorous range (multivariate correlation ∼ 0.57 at > 6 METs, Figure 4A). For relative intensity, lower light intensity was associated with higher CRF, while mid- and upper light intensity showed negative associations. Negative associations peaked in upper light intensity (multivariate correlation ∼−0.71), persisting through lower moderate intensity and diminishing at higher intensities, approaching equilibrium near the vigorous threshold (64% VO_2_max, Figure 4B).

### 3.4 Cumulative cut-point analysis

Cumulative cut-point analysis examined CRF associations using two complementary approaches. First, cumulative correlations (Figures 4C,D) showed how the strength of association between CRF and time above progressively higher intensity thresholds changed across the spectrum. For absolute intensity, correlations increased from mid-moderate intensity upward, peaking in mid-to-upper vigorous range (Figure 4C). For relative intensity, negative associations diminished progressively with increased thresholds, reaching equilibrium in mid-moderate range (Figure 4D).

Second, cumulative regression coefficients (Figures 4E,F) quantified the magnitude of CRF associations, expressed as mL/min/kg per minute of physical activity above each threshold. For absolute intensity, associations increased approximately linearly in moderate-to-vigorous range followed by exponential increase at highest intensities (Figure 4E). For relative intensity, coefficients showed moderate patterns in light-to-moderate range with inverted exponential association at highest intensities (Figure 4F).

## 4 Discussion

This study aimed to characterize how absolute and relative PA intensity relate to CRF in middle-aged women across a wide fitness range. Three primary findings emerged. First, positive associations between absolute PA intensity and CRF began to appear at approximately 4.5 METs and strengthened progressively, peaking in the vigorous range (> 6 METs). Second, the relative-intensity model explained substantially more CRF variance (R^2^ = 0.70) than the absolute-intensity model (R^2^ = 0.53). Third, the inactive group accumulated more relative moderate-intensity time than the active group despite less absolute activity, a clear manifestation of the intensity paradox. Together, these findings have direct implications for accelerometer-based PA surveillance and for exercise prescription in sports science and clinical exercise physiology, suggesting that vigorous intensity should be the primary target when CRF is the desired outcome. Both absolute and relative approaches converge when relative intensity is calculated for average CRF: 4.5 METs corresponds to 46% VO_2_max, and 6 METs to approximately 64% VO_2_max for the sample mean (34.8 mL/min/kg). For typical middle-aged adults with VO_2_max of 33 mL/min/kg, the traditional 3 METs threshold represents only 30% of maximal capacity (1, 3, 6), well below where significant associations emerge.

The inactive group accumulated more relative moderate intensity (36.8 vs. 14.2 min/day, *p* < 0.001) despite less absolute activity. Routine activities such as brisk walking represent a much larger fraction of aerobic capacity, and elicit substantially higher heart-rate responses, in low-fit individuals because their VO_2_max ceiling is lower. The same walking bout that classifies as light relative intensity in a recreational runner therefore reaches the moderate (46%–64% VO_2_max) zone in an inactive woman. Conversely, the active group accumulated more relative light intensity (235.5 vs. 182.4 min/day, *p* < 0.001), because their higher fitness ceiling reclassifies the same walking-equivalent activities into a lower relative intensity category. This is the mechanistic basis of the “intensity paradox” (11), and underscores why surveillance metrics that ignore individual fitness systematically misclassify the aerobic strain of routine activity. The vigorous intensity associations reflect the wide CRF range (20–60 mL/min/kg) and running-focused patterns. This aligns with evidence that vigorous PA explains most variation in CRF improvements and health (5), and that shorter vigorous durations provide greater benefits than longer moderate activity (14). Furthermore, the results support that in previously sedentary middle-aged adults, both higher amount and higher intensity of training are required to maximise CRF gains (26).

Our findings complement research suggesting moderate intensity thresholds of 4–5 METs. Previous individualized calibration studies identified relative moderate intensity (46% VO_2_max) as corresponding to ∼ 4.6 METs (8, 9), matching where positive associations emerge. Cumulative equilibrium at approximately 5 METs supports this moderate threshold for identifying individuals engaging in CRF-related activity, suitable for surveillance. However, associations strengthen progressively with increasing intensity, reaching peak significance in the vigorous range. Regression coefficients quantify this pattern, showing each minute above vigorous thresholds associates with larger CRF differences. In this sample, these data suggest moderate intensity (4.5–5.5 METs) as minimum for surveillance, while vigorous intensity (> 6 METs) provides primary stimulus for CRF maintenance and improvement.

In international reference data for cycle-ergometer cardiopulmonary exercise testing [FRIEND registry (27, 28)], the 50th-percentile VO_2_max for women aged 50–59 and 60–69 years is approximately 21–22 and 17–18 mL/min/kg, respectively. It should be noted that FRIEND is compiled from clinical CPET laboratories and the participants are individuals referred for exercise testing who were subsequently classified as without cardiovascular disease (2017 cohort) or as apparently healthy (2022 cohort), rather than a population-based random sample, which may shift the distribution toward somewhat lower CRF than in purely population-based cohorts. Our overall mean (34.8 mL/min/kg) is markedly higher because approximately half the sample were purposely recruited recreational endurance athletes. Even our inactive subgroup (27.7 mL/min/kg) is above the FRIEND 50th percentile, which we attribute to the exclusion of women with cardiovascular disease, diabetes, cancer or current tobacco use, and to the generally higher population CRF in Sweden (10). The wide CRF distribution that this deliberate recruitment generated (20–60 mL·kg^−1^·min^−1^) is essential for the present analysis: it permits the PA-CRF relationship to be characterised across almost the entire physiological spectrum that would be encountered in a general middle-aged female population, rather than within the narrow range typical of epidemiological cohorts.

The 6 METs vigorous-intensity threshold corresponds to an absolute oxygen uptake of approximately 21 mL/min/kg (6 × 3.5). For this absolute threshold to coincide with the relative vigorous boundary (64% VO_2_max), an individual's VO_2_max needs to be approximately 32.8 mL/min/kg, close to our sample mean (34.8 mL/min/kg). The classical 6 METs criterion is therefore an appropriate vigorous threshold for the present sample, but is too high for women with lower VO_2_max: at the FRIEND 50th-percentile values for women aged 50–69 years (≈ 17–22 mL/min/kg) (27, 28), 6 METs would correspond to roughly 95%–125% of VO_2_max, physiologically unattainable in routine PA. Translated back into absolute units, the relative vigorous boundary (64% VO_2_max) for these women lies at only ≈ 3.1–4.0 METs (e.g., 18 mL/min/kg × 0.64 ÷ 3.5 ≈ 3.3 METs), and the relative moderate boundary (46% VO_2_max) falls at ≈ 2.2–2.9 METs, below the conventional 3 METs absolute moderate threshold. Brisk walking, stair climbing or carrying loads therefore already constitute vigorous relative intensity for the least fit women, while ordinary household ambulation can reach the relative moderate zone. For such low-fit individuals, intensity should accordingly be prescribed relative to individual capacity [e.g., ≥ 64% VO_2_max, ≥ 85% HRmax, RPE ≥ 14, or the qualitative criterion of “substantial increase in breathing rate” (1, 29)]. This is consistent with the intervention evidence from the STRRIDE trial that previously sedentary middle-aged adults gain CRF in a graded manner with both training amount and intensity (26).

The cumulative cut-point and multivariate PLS analyses presented above are two separate analyses applied to answer different questions, and they accordingly identify different equilibrium thresholds. Cumulative analysis identifies the lowest intensity above which time spent is, on aggregate, positively associated with CRF (here ≈ 55% VO_2_max, ≈ 5 METs); it is therefore best suited to surveillance, where the goal is to quantify how much fitness-relevant activity individuals accumulate. Multivariate PLS analysis, by contrast, isolates the intensity range that uniquely contributes to CRF when the entire distribution of activity is accounted for (here ≈ 64% VO_2_max, ≈ 6 METs); this is the more informative threshold for designing interventions or prescribing exercise, because it pinpoints the intensity at which the marginal CRF return is greatest.

PLS regression handled multicollinearity in detailed physical activity data. Traditional regression fails with high-resolution accelerometry due to the closed 24-hour structure (22). Negative associations in light intensity (Figure 3D) despite greater absolute activity (Figure 3B) reflect non-uniform distribution within this category. Time accumulates predominantly in lower light intensity (14%–30% VO_2_max) where negative CRF associations are strongest.

Association strength exceeds previous research, though interpretation requires care given the study design. The relative intensity model explained 70% of CRF variance (R^2^ = 0.70), nearly three times stronger than typical PA-CRF studies (3, 4). The absolute intensity model explained 53% (R^2^ = 0.53), more than double previous findings. These strong associations likely result from diverse CRF range (20–60 mL/min/kg) and maximal exercise testing with validated VO_2_max estimation rather than submaximal predictions, minimizing measurement error while maximizing between-individual variance. The particularly high R^2^ for relative intensity warrants consideration, as it inherently incorporates CRF when calculating intensity categories (% of individual VO_2_max), creating mathematical dependency. However, even the absolute intensity model (R^2^ = 0.53), which has no such dependency, substantially exceeds typical associations, suggesting methodological improvements genuinely enhance detection beyond circularity.

### 4.1 Strengths and limitations

Maximal cycle ergometer testing with validated VO_2_max estimation minimized measurement error compared to submaximal testing, critical for accurate relative intensity calculations. While VO_2_max was estimated from maximal power output using a validated equation rather than measured directly through gas analysis, this approach provides substantially more accurate assessment than submaximal testing, particularly at higher CRF levels where submaximal predictions are least reliable. The diverse CRF range (20–60 mL/min/kg) enabled examination across the physiological spectrum (3, 4). The detailed intensity spectrum analysis using 4 Hz frequency extended accelerometry captures high-intensity activities more accurately than traditional methods (19). The cumulative cut-point analysis provided both statistical significance and practical relevance.

The relatively small sample (*n* = 73) and specific population (middle-aged women) limit generalizability. Population studies suggest age- and sex-specific approaches may be necessary (13, 14). The 7-day accelerometry period may inadequately capture sporadic vigorous activities in the inactive group, though consistent patterns across analytical approaches suggest robust findings (30). The cross-sectional nature presents interpretive challenges, particularly regarding relative intensity. In established activity patterns, PA, CRF, and health outcomes have likely reached equilibrium, making relative intensity less informative than in intervention contexts (12). However, the convergence of absolute (4.5–5.0 METs) and relative (46% VO_2_max for average CRF) thresholds provides practical guidance transcending these limitations.

Beyond the demographic profile, two further boundary conditions limit generalizability. First, the sample was recruited from one Swedish region and we did not collect detailed data on socioeconomic status, education or occupation; PA-related health gains in midlife differ markedly across socioeconomic strata (31). Second, we deliberately excluded participants with established cardiovascular disease, diabetes, cancer or current tobacco use, so the findings cannot be extrapolated to middle-aged women with these conditions, in whom both the absolute level and the trajectory of CRF differ from healthy peers (32).

A key methodological limitation is that VO_2_max was estimated from maximal power output rather than directly measured through gas exchange, which may introduce some error in relative intensity categorization despite the validated protocol and high test-retest reliability (r = 0.95–0.96). While the prediction equation (18) was originally validated in younger adults (15–28 years), the linear relationship between maximal power output and VO_2_max is well-established across age groups (16, 17), and the use of maximal rather than submaximal testing reduces age-related prediction error. Nevertheless, individual deviations from the population-average power-VO_2_ relationship could shift relative-intensity classifications by approximately one bin. This would attenuate the clarity of inter-group differences but is unlikely to change the direction of the reported associations.

### 4.2 Implications

These findings have important implications for exercise prescription and public health. Vigorous intensity (> 6 METs, > 64% VO_2_max) shows the strongest associations with CRF. This indicates exercise guidelines may need to place more emphasis on vigorous activity as optimal for CRF maintenance and improvement. Current moderate intensity recommendations (3 METs) are insufficient, but even the revised moderate threshold (4.5–5.0 METs) represents a minimum standard rather than optimal target. For maximizing CRF-related health benefits, vigorous intensity appears to provide the primary stimulus.

Vigorous intensity can be prescribed using relative measures accounting for individual capacity. Rather than prescribing specific activities, practitioners should target intensities resulting in “substantial increase in breathing rate” (1, 29), using perceived exertion scales or heart rate monitors (> 64% of maximum). This naturally adjusts for CRF level: brisk walking may represent vigorous intensity for deconditioned individuals, while running at higher intensities would be required for fitter individuals. The moderate threshold (4.5–5.0 METs, 46% VO_2_max) remains relevant as minimum standard for population surveillance but may not represent the optimal target.

For population health, these findings support updated surveillance methods considering CRF-adjusted thresholds. Recent age- and sex-specific accelerometer cut-points represent movement toward this approach (13, 14). However, individualized CRF-adjusted thresholds require knowledge of VO_2_max, which limits direct population-level application. Age- and sex-specific accelerometer cut-points (13, 14) offer a practical compromise, approximating relative intensity without individual CRF testing. Previous mediation analysis showing 82% of PA-health associations operate through CRF improvements reinforces these findings (5). If health benefits primarily occur through CRF adaptations, prescriptions should target intensities sufficient to improve CRF, which occurs primarily at vigorous intensity.

### 4.3 Future research directions

Longitudinal studies examining how changes in relative intensity predict CRF improvements and health outcomes are essential for establishing causality (12). Investigations of whether CRF-adjusted intensity thresholds improve health outcome prediction beyond absolute measures would provide crucial evidence for clinical implementation. Research across diverse populations, particularly older adults and those with chronic diseases, would inform broader applicability (15).

## 5 Conclusions

This study demonstrates that, in this sample of middle-aged women with a wide CRF range, traditional absolute moderate intensity thresholds (3 METs) appear inadequate, with significant CRF associations occurring primarily at vigorous intensity (> 6 METs, > 64% VO_2_max). While positive associations begin emerging at mid-moderate intensity (∼ 4.5 METs, ∼ 46% VO_2_max), strength and consistency increase progressively, reaching peak significance in the vigorous range. This reflects the principle that health-beneficial physical activity intensity must be relative to individual physiological capacity, with vigorous relative intensity providing primary stimulus for CRF-related health benefits. Guidelines should emphasize that vigorous intensity provides optimal stimulus for CRF maintenance and improvement, while moderate intensity represents the minimum threshold where associations emerge.

## Data Availability

The datasets presented in this article are not readily available because of participant privacy restrictions. Requests to access the datasets should be directed to jonatan.fridolfsson@gu.se.
